# Religious moderation in Instagram: An Islamic interpretation perspective

**DOI:** 10.1016/j.heliyon.2025.e42816

**Published:** 2025-02-19

**Authors:** Andy Hadiyanto, Kinkin Yuliaty Subarsa Putri, Luthfi Fazli

**Affiliations:** a Department of Islamic Education, Faculty of Social Sciences, Universitas Negeri Jakarta, Indonesia; b Department of Communication Science, Faculty of Social Sciences, Universitas Negeri Jakarta, Indonesia

**Keywords:** Religious moderation, Social media, Islamic interpretation, Pluralism, Instagram

## Abstract

Religious freedom and plurality remain major challenges in Indonesia, with both authorities and social media influencers involved. One potential solution is integrating moderation into religious activities, especially through platforms like Instagram. Radicalism in Indonesia, particularly among youth, is fueled by extremist narratives, events like the 2018 Surabaya bombings, and the return of ISIS recruits. This highlights the need for research into religious moderation and extremism prevention. The Surabaya bombings, which targeted civilians, and the return of around 1,000 Indonesians who joined ISIS have escalated the threat of radicalization. Surveys show that about 7.7 % of Indonesians, or 600,000 people, are open to radical activities, making it urgent to address radicalism with religious moderation as a counter-narrative. This study focuses on religious moderation in Qur'anic interpretation and explores the role of social media in promoting moderation. Instagram's effectiveness in encouraging religious moderation is analyzed using a descriptive-qualitative approach, investigating various sources and publications. Instagram promotes a more inclusive and harmonious society by fostering tolerance, diversity, and mutual respect. The study highlights how Instagram's religious moderation initiatives provide balanced perspectives across different beliefs, helping to spread understanding and combat extremism. With its broad reach, especially in Indonesia, Instagram is an effective tool for promoting messages of moderation and supporting diversity and peace.

## 1 Introduction

**Fig. 1:**
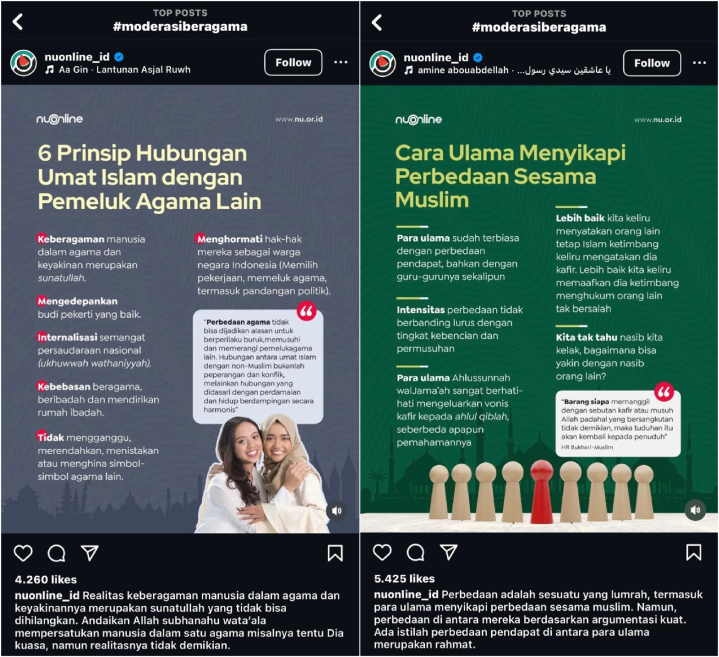
Religious moderation movement campaign forms on Instagram.

The complexity of religious diversity and beliefs in today's global order has increased with the progress of globalization, necessitating the challenge of maintaining harmony and inter-religious harmony within the framework of life in order to practice religion in accordance with each individual's beliefs [1]. Given this, religious moderation becomes an important solution that merits further examination, particularly in light of the countless conflicts, prejudice, and intolerance that develop as a result of the concept's lack of general adoption [2]. Unfortunately, issues that undermine tolerance within religious communities or among individuals of the same faith constantly plague Indonesia. Additionally, Indonesia's religious diversity is consistent with the teachings of the Prophet Muhammad SAW, specifically “*rahmatan lil ‘alamin*,” which highlights the importance of studying and putting into practice religious moderation from an Islamic perspective for all Indonesians [3] (see Fig. 1).

Religious moderation entails minimizing violence and avoiding fanaticism. Religious moderation can encompass a moderate perspective of variety. This is an effort to accommodate Indonesia's diverse religions. Religious moderation involves allowing space for religions that adhere to absolute teachings and are widely held by others. Religious moderation refers to taking a neutral or middle-ground posture between opposing perspectives. Encourage active listening and practice overcoming differences. Religious moderation signifies an endeavor by society to accommodate the multiplicity of religions in Indonesia. Religious moderation seeks to eliminate violence and prevent radicalism [4]. It can also be construed as a moderate or neutral attitude toward the multiplicity of religions or beliefs practiced by the majority of society. As a result, the foundation of religious moderation is to respect other people's religions while adhering to absolute religious principles [5].

Radicalism is a major concern in Indonesia, particularly among young people and in educational institutions. Extremist narratives drive religious extremism on major campuses, prompting stakeholders to recommend a more integrated approach to effectively address this trend. The growth of groups such as Jama'ah Anshorut Daulah (JAD) and Jama'ah Anshorut Tauhid (JAT), which have sworn allegiance to the Islamic State, highlights the ongoing threat of violent extremism, including noteworthy assaults such as the Surabaya bombings of 2018. Around 1,000 Indonesians traveled to Syria to join ISIS, with many coming home, increasing the risk of domestic radicalization.

Religious moderation can also refer to a viewpoint or attitude that seeks to be neutral between two existent points of view, as well as an attitude that strives to respect one another and create knowledge or the ability to overcome differences [6]. Religious moderation promotes equity and balance in religious practice while avoiding human hedonism, extremism, and revolutionary religious attitudes [7]. Religious moderation can help Indonesians enhance their religious diversity. As a consequence, the cultural factors of Indonesia's pluralistic society are well suited for application in Indonesia.

The country is an archipelago with a diverse society comprised of many tribes, races, ethnicities, and faiths, with over 600 tribes. Every individual has the right to express their thoughts and themselves in public while emphasizing truth, honesty, and facts. Legislation protects the freedom of speech, according to Article 29 of the Universal Declaration of Human Rights and Article 28 of the 1945 Constitution. According to Article 29 of the 1945 Constitution, the state is based on the Almighty God and guarantees the freedom of every citizen to embrace religion and worship according to their beliefs. Misconceptions about religion with regard to worship remain entrenched and difficult to change. The excessive fanaticism that has recently become common can lead to individuals ignoring government regulations and even vandalism, which ultimately can trigger less commendable actions and potentially divide unity in Indonesia.

Indonesians have long practiced religious moderation. It is evidenced by the presence of recognized beliefs in Indonesia, and all adherents understand the meaning of religious moderation, just as in Islamic teachings, namely by the term *washatiyah*, which has the equivalent meaning or definition as *tawasuth*, which means the middle, *i'tidal*, which means fair, and *tawazun*, which means balanced [8]. Some interpretations argue that Protestants provide answers in their teachings that only a few understand. However, the Catholic Church does not regard religious moderation as anti-modern or prone to fundamentalism and traditionalism. In truth, the Catholic Church defines religious moderation as a balanced approach that combines essential religious beliefs with modernity, emphasizing social justice, human dignity, and involvement with contemporary society. The Church's position on religious moderation is centered on promoting communication with the modern world while maintaining its essential doctrines [9].

In the context of interfaith relations, the Catholic Church emphasizes the significance of constructive dialogue as a core aspect of religious moderation. Documents such as *Nostra Aetate* underscore the Church's recognition that all religions contain positive values that can enrich humanity universally. The Catholic approach to religious moderation seeks to balance core doctrines with contemporary values while promoting social solidarity in addressing global challenges such as poverty, injustice, and radicalism. This perspective highlights the importance of respecting religious diversity as a foundation for fostering harmony and constructive cooperation among different faith communities [9].

Christianity also offers valuable answers to the discourse on religious moderation through its principles of universal love and forgiveness, which are often misunderstood. For instance, Jesus Christ's teaching of unconditional acceptance, even of those with differing beliefs, exemplifies the practice of harmonious coexistence. The radical nature of forgiveness, as seen in the command to “love your enemies,” provides a profound foundation for overcoming divisions. Additionally, the concept of self-sacrifice, as embodied in the crucifixion, serves as a model for solidarity and devotion to the common good, offering a transformative perspective on the role of faith in building societal harmony.

In the Indonesian context, characterized by its extensive religious diversity, the perspectives on religious moderation from both Catholic and Christian traditions hold particular relevance. The emphasis on human dignity and freedom of religion aligns with the need to prevent conflict and foster social harmony. Moreover, Islamic teachings, such as *rahmatan lil ‘alamin*, share a common foundation with Christian values in promoting moderation, tolerance, and interfaith dialogue. By strengthening these shared principles, Indonesia can address the challenges of radicalism while reinforcing unity and mutual respect among its diverse communities.

Aside from that, the most fundamental teaching of Hinduism in religious moderation is susila, or how attempts to develop harmonious relationships between humans are among the three causes of well-being. Sidharta Gautama, a Buddhist figure, explains religious moderation. Sidharta Gautama established four *prasetya*; (1) serve others; (2) reject all worldly pleasures; (3) study and practice the *dharma*; and (4) strive for perfect enlightenment [10]. Another issue is that the Confucian religion's teachings instill in its followers the virtue of viewing life in terms of *yin* and *yang,* because *yin* philosophy is the ideology and spirituality of Confucian believers who strive to live in the *Dao*. The *yin* and *yang* symbolize completion rather than excess [11].

Despite Indonesia's current security and tranquility, the necessity for religious moderation remains critical. While frequently regarded as peaceful, underlying tensions and the possibility of radicalization continue, notably through social media sites such as Instagram, which can transmit extremist messages to vulnerable youth. The problem of people flying to conflict zones to join extremist groups highlights the dangers of domestic radicalization. As a result, creating an environment of tolerance and understanding is critical for maintaining social harmony in Indonesia's diverse population. Implementing initiatives like institutionalizing religious moderation in policy, improving community education, and incorporating these efforts into national development plans will assist to create a more inclusive society and avert future disputes [11].

Although Indonesia is currently in a safe and peaceful condition, the threat of radicalization remains present, particularly through social media platforms like Instagram. Research indicates that Instagram is utilized by extremist groups to disseminate radical narratives through visual posts, provocative videos, and the strategic use of hashtags that attract the attention of vulnerable groups, especially young people. Data shows that approximately 7.7 % of Indonesia's population is willing to engage in radical activities, highlighting a significant potential for domestic radicalization. Incidents such as the 2018 Surabaya bombings and the return of ISIS recruits to Indonesia further underscore the dangers of online-fueled radicalism [10]. In this context, religious moderation is not only a necessity but also a crucial strategy to create spaces for dialogue and tolerance, effectively countering the spread of extremist ideologies. Strengthening moderation through policies, community education, and integration into social media platforms like Instagram represents concrete steps to mitigate the threat of radicalization amidst Indonesia's diverse society.

Religious moderation is not merely an option but a necessity for maintaining social harmony in a diverse nation like Indonesia. It plays a vital role in promoting good relationships among different communities and ensuring socio-political stability. The educational sector is particularly crucial in instilling values of moderation among students to combat extremism effectively. By promoting initiatives focused on tolerance and respect for diversity, Indonesia can create a more inclusive society. In summary, while the country may currently enjoy relative peace, the ongoing challenges posed by radicalism underscore the importance of implementing religious moderation as a proactive measure to ensure lasting harmony and prevent future conflicts.

In order to improve religious moderation, Indonesian society can employ three major strategies: (1) institutionalizing religious moderation in binding policies and programs; (2) spreading understanding, ideas, and education about religious moderation throughout the community; and (3) incorporating the formulation into the National Medium-Term Development Plan (RPJMN) for 2020–2024. If the year-to-year plan is effective, it can be repeated for the next year and included in the National Medium-Term Development Plan (RPJMN) [12]. The religious moderation movement's techniques can be applied by all Indonesians by spreading understanding, ideas, and education about religious moderation through religious moderation movement initiatives. The campaign can be carried out using social media [13].

Nowadays, social media provides an open platform for the entire global community, particularly Indonesians, to get to know one another and learn about religion. Most individuals utilize social media, which is an information technology invention. The use of social media allows individuals to surf for specific information in a very adaptive and efficient manner [14]. As a result, the religious moderation movement's campaign must be implemented in a coordinated and large-scale manner, particularly using Instagram, Indonesia's most popular social media site and an ideal instrument. The use of Instagram as a marketing tool for religious moderation is expected to be well embraced by all Indonesians [15].

Instagram is the most widely used social media network in Indonesia right now, making it the ideal medium for implementing the campaign for religious moderation in a coordinated and huge manner [16]. All Indonesians are expected to respond favorably to the use of Instagram as a campaign tool for religious moderation. Instagram, with its visually appealing content and large user base, provides a unique chance to effectively communicate messages of moderation and understanding. The platform enables users to interact with content that promotes peace, tolerance, and respect for diverse views, making it an appropriate medium for religious moderation campaigns.

The increasing complexity of religious diversity in today's globalized world necessitates efforts to maintain harmony and tolerance, particularly in nations like Indonesia where religious plurality is firmly ingrained. This situation emphasizes the necessity of religious moderation as a response to long-standing difficulties like prejudice, intolerance, and conflicts that undermine societal cohesiveness.

Indonesia has the world's largest Muslim population, which is a significant concern in Islamic moderation. Moderate Islam is acknowledged to have implications for diversity in a variety of areas, including religion, culture, ethnicity, and nation [17]. However, some Muslims are radical and rigorous in their interpretation of Islamic teachings, even resorting to violence to impose their beliefs [18]. For this reason, researchers are interested in examining the idea of religious moderation, which must be understood contextually rather than merely textually, using the popular social media platform Instagram.

Religious moderation in Indonesia is about building a moderate interpretation of religion in light of the country's diverse culture and customs, rather than moderating the country itself [19]. As a consequence, the researcher proposes a socialization content style that incorporates concepts, understanding, and education about religious moderation, as well as being effective and relevant for campaigning the religious moderation movement on social media, particularly Instagram.

## 2 Literature review

### 2.1 Islam and religious moderation

The Arabic word “*alwasathiyyah*” is derived from the words “*al-wasth*” and “*al-wasath*,” both of which are masdar versions of the verb “*wasatha*.” “*Alwasathiyyah*” derives from the root word “*wasath*.” Moderation is known as “*alwasathiyyah islamiyah*” in Islamic teachings, which refers to a moderate and balanced attitude in religious practice with no leaning to the right or left [20]. Yusuf Qardhawi proposed that theological moderation expresses a realistic view of human nature, following the middle road between two extremes and not taking sides in a certain paradigm. Moderation, in its numerous interpretations, highlights the significance of preserving balance or moderation in behavior, morals, and attitudes when engaging with individuals or state organizations [21].

*Alwasathiyyah*, also known as religious moderation, refers to the core and content of religion that lack excessive aspects in attitudes, ideas, or beliefs [22]. Thus, Yusuf Qardhawi defines alwasathiyyah, also known as *al-tawazun*, as balancing two conflicting viewpoints so that neither is dominant. Religious moderation can be defined as a manner of seeing, acting, being balanced, and not exaggerating religious things. According to the hadith of the Prophet Muhammad SAW, “The best of affairs is *awsathuha* (the middle)” [21]. As a result, Islamic teachings are free of extremism and radicalism since they emphasize balance and justice. Islam emphasizes balance and justice, which are antithetical to extremism and radicalism.

The history of religious moderation in Indonesia began when Islam first arrived in the country. Nadhlatul Ulama and Muhammadiyah are two Islamic groups well-known for their commitment to religious moderation. These two organizations serve a vital role in preserving, protecting, and advancing moderate principles in religious life [23]. Nadhlatul Ulama benefits from the Ahlussunnah wal Jamaah creed's moderate attitude as the primary underpinning for daily living. Meanwhile, Muhammadiyah is well-known for its capacity to balance religious teachings with contemporary changes, establishing itself as a progressive and active organization in Islamic renewal [24].

Both organizations promote moderate religious values, especially among Indonesians, with the aim of improving morals, worship, and faith. The concept of moderation in religion has been upheld by previous figures such as the Prophet, companions, and scholars who always showed a fair and balanced attitude towards others, regardless of ethnic, racial, linguistic, or religious backgrounds.

To address the concern about the proper explanation of data on the spread of radicalism on Instagram, it is essential to explore the specifics of how extremist ideologies are disseminated through this platform. Radicalism in Indonesia is often propagated via social media, particularly Instagram, which is widely used by approximately 85 % of Indonesian social media users. This makes Instagram a significant channel for reaching a broad audience with radical content. Extremist accounts utilize various mechanisms, including provocative posts, videos, and resonant hashtags, to influence vulnerable groups and promote their ideologies.

Research indicates that these accounts often employ visual content that opposes moderate values, thus shaping public perceptions and encouraging recruitment. A notable example involves selected Instagram accounts focused on de-radicalization efforts that actively engage in media literacy by sharing narratives against radicalism and violence. However, there remains a gap in quantitative analysis regarding comment responses to such campaigns, highlighting the need for further research that includes multiple social media platforms beyond Instagram.

Expanding the scope of research could provide insights into how different platforms affect the dissemination of radical versus moderate content, ultimately aiding in the development of targeted strategies to mitigate extremism. By integrating these observations with the historical context of religious moderation in Indonesia, the urgency of promoting balanced religious practices becomes evident. Organizations like Nadhlatul Ulama and Muhammadiyah have long championed moderate Islamic values, serving as models for balancing traditional teachings with contemporary realities. Understanding the mechanisms driving radicalism on Instagram is crucial for designing effective counter-narratives that promote religious moderation and inclusivity, aligning with Islamic teachings that emphasize balance and justice over extremism and radicalism.

The significance of Instagram in promoting radicalism demands more information and explanation. According to research, extremist themes thrive on social media, especially among young people, making Instagram an important venue for radicalization in Indonesia. The 2018 Surabaya bombings and the return of ISIS recruits emphasize the menace of extremism. To back up these allegations, concrete data points and examples should be offered, such as screenshots and links to studies that show how extremist groups use Instagram to spread their messages. This holistic strategy will enhance the case for religious moderation while emphasizing the significance of effectively combating radicalism in Indonesia [24].

### 2.2 The meaning and values of religious moderation

#### 2.2.1 The meaning of religious moderation

According to Ismail Raji (1986), religious moderation is balance (*tawazun*), also known as “the golden mean,” which is an attitude that seeks a balanced middle ground rather than two extremes. Furthermore, Ragib Al-Ashfahani (502 Hijriyah) also defined moderation as being in the middle, not leaning to the left or right, but balanced by favoring nobility, equality, and justice [25]. Approaches to supporting and promoting religious moderation need to involve tolerance, mutual respect, and understanding between different religions and their adherents. This includes recognizing and respecting the diversity of religious beliefs and promoting interfaith dialogue, cooperation, and peace [26]. The basic principles of religious moderation include respect, tolerance, interfaith dialogue, cooperation, and understanding. Religious moderation aims to create an inclusive society that respects differences and religious freedom. However, extremism, intolerance, and conflict due to religious differences often pose challenges to religious moderation [27].

#### 2.2.2 The values of religious moderation

Generally, religious moderation consists of four main indicators; moderation in religion (*tawasuth*), tolerance of differences (*tasamuh*), maintaining balance in various aspects of life (*tawazun*), and consistency and justice in action (*i'tidal*) [28]. Moreover, the concept of *wasathiyyah* also includes nine other characteristics, including: taking a moderate attitude in religion (*tawasuth*), distinguishing between differences and deviations (*tawazun*), being firm and fair in carrying out rights and obligations (*i'tidal*), respecting differences in both religious and life aspects (*tasamuh*), adhering to egalitarian principles without discrimination (*musawah*), problem solving through deliberation (*shura*), reforming for improvement (*islah*), prioritizing important things (*aulawiyah*), and being dynamic and innovative for positive change (*tathawwur wa ibtikar*) [29]. As a result, Islam's teachings provide Indonesians with principles of religious moderation. To preserve harmony between religious communities, the ideals are supposed to be used in the lives of the nation and state. It is consistent with the Pancasila philosophy of omnipotent divinity found in Indonesia [30].

### 2.3 Islam and religious moderation challenges in Indonesia

According to a report from the Royal Islamic Strategic Studies Center (RISSC), Indonesia is the most populous Muslim country in the world, a fact that highlights the importance of moderation in Islam. The concept of moderation is the essence of Islamic teachings that emphasize relevance in dealing with diversity in all areas of life, including culture, religion, ethnicity, and the nation itself [31]. Muslims face increasingly complicated concerns that extend beyond theological issues and into political and social dimensions. Islamic moderation emerges as a new paradigm for viewing religion, embracing the virtues of tolerance, pluralism, and social unity [32]. Therefore, Islamic teachings are beneficial to people's unity, civilizational development, and humanity. The rapid development of Islamic science is expected to prevent conflicts that could harm and tarnish Islam's dignity, as well as to demonstrate that Muslims are continuing to advance in science and morality [33].

### 2.4 Religious pluralism

Indonesia has a diversified society. Furthermore, any religious division or institution, including educators, should examine other religious groups before building its own culture and theology in order to foster contextual understanding and avoid confrontation [34]. The current era recognizes that every religious division or organization must create a curriculum that educates people by removing the duality of religious education from each religion and reviewing every doctrine to comprehend the substance of contextual teaching [35]. Indonesia is also renowned as an archipelago with abundant natural resources and ethnic, cultural, religious, racial, and linguistic diversity; therefore, it is full of contrasts from numerous perspectives [36].

Religious pluralism is one of Indonesia's defining characteristics. Etymologically, the term comes from the words “plural,” which means many, and “ism,” which means sect or belief. Religious pluralism refers to the coexistence of different religions without losing the uniqueness of each. It emerged because of the one-sided truth claims of each religion, which often considered other religions heretical, triggering conflicts, wars, and oppression between citizens. The cause of these conflicts is the emergence of religious pluralism. Religious pluralism considers all religions equal without any one being superior. The goal is to create harmony in diversity [37].

Pluralism, a Western concept based on relativism, emphasizes that there is no absolute truth, including in religion. It follows that no one has the right to judge the beliefs of others, and within heterogeneous societies, all religions are considered equal. Religious exclusivity often triggers conflict, humiliation, and persecution. The view that only certain religions deserve to lead the country is characteristic of the adherents of exclusivity or fundamentalism [38].

### 2.5 Religious moderation based in the Qur'an and Hadiths

#### 2.5.1 QS Al-Baqarah: 143

Religious moderation in Islam should first be grounded in the Qur'an, which emphasizes the importance of a balanced approach. For instance, Allah describes the followers of Prophet Muhammad as a moderate community (*wasatha*) in Surah Al-Baqarah: 143. This foundational principle underscores that moderation is not merely an option but a divine mandate for Muslims. Following this, hadith can be referenced to further illustrate the concept of moderation. The hadith stating, “The best of affairs is *awsathuha* (the middle),” reinforces this idea, showing that Islamic teachings inherently promote balance and justice, countering extremism and radicalism [39].

#### 2.5.2 Sahih Al-Bukhari

According to Abu Hurairah, the Messenger of Allah declared, “*A person's deeds will not save him*.” “*Did you too, Messenger of Allah?*” one of them then inquired. “*And so am I*,” the Prophet retorted. “*But if Allah SWT.* grants *His favor, then straighten your intentions and let you not feel excessive* (in charity that makes boredom). *Hurry in the morning and afternoon, and do it also at the end of the night*.” To get there, you have to walk in the middle. (Muhammad bin Isma'il bin Ibrahim bin al-Mughirah Abu ‘Abd Allah al-Bukhari, n.d.) [39].

#### 2.5.3 Sahih Muslim

“*I was with the Prophet Muhammad SAW, praying several times together, and (I have found) the middle in the prayers and the sermons as well*” stated Jabir bin Samurah. (Muhammad bin. Isma'il bin Ibrahim bin al-Mughirah Abu ‘Abd Allah al-Bukhari, n.d.) [39].

#### 2.5.4 Sunan an-Nasai and Ibn Majah

“*Allah's creation, avoid being excessive (exceeding the limit), because the people before us were destroyed and perished by crossing the limit in religion*,” stated the Prophet Muhammad SAW, as quoted by Ibn ‘Abbas (al-Nasa'i, n.d., Al-Qazawayni, n.d.) [39].

Prioritizing the source of religious moderation in the Qur'an is essential before referencing hadith. The Qur'an serves as the foundational text that emphasizes the principle of moderation, known as “*wasathiyyah*,” which promotes a balanced approach to faith and practice. This principle is reflected in various verses that encourage Muslims to avoid extremism and adopt a middle path in their beliefs and actions. By grounding discussions of religious moderation in the Qur'an, a more robust understanding of its core values can be established, providing a solid foundation for further exploration of hadith that complements these teachings.

The hadith can then be utilized to illustrate specific applications of moderation in the lives of the Prophet Muhammad and his companions, reinforcing the Qur'anic teachings. For example, sayings attributed to the Prophet emphasize avoiding excess and maintaining balance in worship and daily conduct. By first establishing the Qur'anic basis for religious moderation, subsequent references to hadith can enhance the discussion, demonstrating how these principles were lived out historically. This approach ensures a comprehensive understanding of religious moderation that is deeply rooted in Islamic teachings.

### 2.6 Social media

The word “medium,” which means “mediator or intermediary,” is pluralized as “media.” Specifically, media serves as a tool for communication when it comes to information sharing. Telegram, Facebook, Instagram, WhatsApp, YouTube, and other internet-based communication platforms are examples of social media. The larger community can self-actualize, connect with others, and seek and provide information thanks to social media. Social media is one type of intermediary used in communication [40]. Along with the development of technology, social media has become a primary need in human life as it is efficient and effective for communication and access to information. Instagram is one of the most popular social media platforms in Indonesia. The latest report from We Are Social shows that Instagram takes second place as the most used social media app in Indonesia, with 85.3 % of users, followed by Facebook (81.6 %) and TikTok (73.5 %). Instagram is considered to be very informative and a means of expression [41].

### 2.7 The urgency of implementing religious moderation in social media

Around 600,000 Indonesians, or 7.7 % of the country's population, are willing to carry out radical activities, according to a 2016 assessment by the Wahid Foundation. People who are not radicalized might be negatively impacted by the number of intolerable individuals who adhere to religious moderation. Access to the internet can lead to a rise in radical attitudes and perspectives through social media, particularly when it comes to intolerable content [42].

Because of its widespread reach, social media has become an essential aspect of people's lives. Social media has the power to shift people's perspectives and make jobs easier. People's perspectives on key problems can be influenced by widely available information on social media platforms [43] For example, knowledge regarding the COVID-19 virus, which was reactively responded to by the public, caused the price of masks and face shields to rise from Rp45,000–Rp60,000 to Rp350,000–Rp2,500,000 due to bulking and hoarding. This made masks and face shields difficult to come by during the COVID-19 epidemic in 2020–2022. The social media structure can thus build the user's reality based on the content of the media ingested, because the media can influence a person's actions based on the impressions perceived [44].

Social media has unquestionable power as a platform for influencing public opinion on an issue. Social media has heightened debates about Islamic discourse, religious movements, and Muslim relations and manifestations. Social media's features, such as real-time engagement, sharing, and archiving, make it a vehicle for amplifying Islamic concerns. According to the Digital Report survey, the usage of social media in Indonesia is expanding dramatically, with changes in the popularity of social media platforms, particularly Instagram [45]. It is not surprising that the discourse of religious moderation has also been amplified on social media to account for the narratives of radicalism, terrorism, and intolerance that have participated in the conflict and seized the public opinion of Indonesian citizens.

In the 21st century, religious discrimination still occurs frequently in Indonesia, especially through hate speech on social media. The research by Hastak & Risal (2021) shows that the misuse of social media by irresponsible people can divide the unity of the Republic of Indonesia. These activities include discrimination, intolerance, and various forms of hate speech against certain religions. Facing this phenomenon, there is a need for a new counter strategy, namely efforts to promote religious moderation by utilizing social media [46].

Issues of radicalism, intolerance, and religious conflict are unjustified and detrimental to Indonesian society. Promoting religious moderation through social media is essential for safeguarding harmony and peace within the nation. Consequently, it is crucial for individuals to use social media thoughtfully and effectively, preventing the spread of extremist ideas while highlighting the benefits of a moderate approach to religion [47]. Platforms like Instagram have emerged as powerful tools for fostering religious moderation movements. By leveraging visual content and interactive features, Instagram can engage users in discussions that promote tolerance and understanding among diverse religious groups.

The widespread use of social media enables messages of moderation to reach a broader audience, encouraging a collective effort to counteract radical ideologies. Long-term engagement on social media platforms can facilitate the implementation of religious moderation, allowing Indonesian society to work together in preventing interfaith conflicts. By actively participating in moderation campaigns on Instagram, users can contribute to a culture of respect and cooperation, ultimately enhancing social cohesion and fostering a peaceful coexistence among various faith communities.

The urgency of implementing religious moderation in social media, particularly on platforms like Instagram, is heightened by the concerning spread of radicalism. For instance, extremist narratives have proliferated on Instagram, influencing vulnerable youth and potentially triggering real-world acts of radicalism. This dynamic is exemplified by incidents such as the 2018 Surabaya bombings, which were linked to individuals radicalized online. Therefore, it is crucial to enhance educational initiatives that promote moderation and tolerance within these digital spaces. Coordinated efforts to counteract extremist content on social media can help build a more inclusive society and prevent the escalation of radical ideologies into violent actions.

The influence of social media extends beyond mere opinion formation; it can distort reality and provoke irrational behavior. For instance, during the COVID-19 pandemic, misinformation circulated rapidly on social platforms, leading to panic buying and inflated prices for essential items like masks and face shields. In Indonesia, the proliferation of hate speech online exacerbates radicalism, contributing to social division and discrimination.

In response to these challenges, promoting religious moderation through social media emerges as a vital counterstrategy. Platforms like Instagram can facilitate discussions that emphasize tolerance and understanding among diverse religious communities. By leveraging visual content and interactive features, these campaigns can effectively engage users, fostering collective efforts to combat radical ideologies while enhancing social cohesion and peaceful coexistence.

## 3 Methods

The theoretical framework concerning the relationship between religious moderation and social media is underdeveloped and lacks comprehensive analysis. The study employed a qualitative methodology with a descriptive-qualitative approach, allowing researchers to delve into facts and concepts related to religious moderation. However, the article offers only a brief description of this methodology, failing to elaborate on how the data was collected and analyzed. The primary goal of the qualitative method is to present a thorough exposition of findings, ideas, and solutions through extensive examination.

Qualitative methods are particularly effective in social research as they describe, summarize, and explain relevant situations or variables that resonate with the general public [48]. These methods are widely used in the social sciences and humanities to gain insights into patterns, human behavior, and aspects that are difficult to quantify. By emphasizing inductive reasoning, qualitative research relies on participatory observation of social processes [49]. This approach provides a richer understanding of the complexities surrounding religious moderation and its expression through social media, thus addressing the theoretical gaps in existing literature while also highlighting the need for a more detailed methodology description.

The theoretical framework concerning the relationship between religious moderation and social media is notably underdeveloped and lacks in-depth analysis. This study aims to investigate, from an Islamic perspective, how religious moderation is practiced on social media by evaluating various documents, including books, research reports, and articles from scientific journals. The primary objective of the literature review in this qualitative study is to characterize and clarify the key terms used. While qualitative communication research does not focus on testing hypotheses, researchers are not confined to specific definitions; however, they must apply relevant theories and possess a solid understanding of the concepts being explored.

Despite this flexibility, the researcher relies on a comprehensive grasp of the underlying principles. Therefore, this study employs a literature review and documentation as its two main methods of data collection. By investigating the impact of Instagram content on Indonesia's religious moderation campaign, specifically analyzing photos and videos tagged with #moderasiberagama, this research effectively establishes strategies for utilizing Instagram as a medium to promote religious moderation through the examination of relevant literature and Instagram content.

## 4 Results

### 4.1 The role of social media as a campaign strategy for the religious moderation movement

Some overgeneralize by making broad claims about social media's influence on public opinion without providing specific evidence or examples to support these assertions. For instance, while it suggests that social media can spark large-scale movements, it does not delve into particular cases or studies that illustrate this phenomenon. Additionally, the article could be strengthened by engaging with opposing perspectives or critiques of religious moderation, particularly in the context of social media. Some critics argue that social media can also amplify extremist views and misinformation, undermining the very moderation it aims to promote.

The rise of new media, particularly digital communication technologies, has been facilitated by advancements in information technology infrastructure. Social media has emerged as a transformative medium, shaping public discourse and influencing how people think by providing a platform for information exchange [45]. This shift has fostered a culture of communication where interactions occur not only face-to-face but also through digital channels [50]. In Indonesia, social media plays a significant role in deepening religious understanding across various platforms. Its accessibility, speed, interactivity, and extensive reach make it a more effective tool than traditional media. Users actively create and share content, facilitating communication and engagement [41].

Campaigns promoting religious moderation heavily rely on social media, leveraging its ability to capture users’ attention and organize public opinion. However, the impact of social media on attitudes and behaviors towards diversity needs further exploration, as it may also serve to amplify divisive narratives. Thus, while social media holds the potential to promote religious moderation, a nuanced understanding of its role, including both its benefits and drawbacks, is essential for developing effective strategies in this area.

## 5 Discussion

### 5.1 Forms of religious moderation campaign on Instagram

Instagram is a popular social media platform on the internet that has attracted widespread interest from all walks of life. Through various features, such as the ability to freely upload photos, videos, and livestream, Instagram gives users a wide range of access [51]. Similarly to other apps such as Facebook, Instagram also allows users to use hashtags to make their posted content easily discoverable by many other users. Instagram provides various important features, such as the hashtag feature marked with the “#” symbol. The feature available on Instagram aims to group content based on certain themes or topics in order to organize the content and make it easier to find by using hashtags on posts [52].

Therefore, to be popular on Instagram, there are several factors that need to be considered, such as high-quality photos or images, clear videos without blurring, captions that describe the content well, and the use of relevant hashtags to reach a wider audience. These factors have a significant influence on the number of likes, comments, and reach of the content. The more users share positive and useful content, the more likely it is to gain positive responses from other users in the form of likes and comments [53].

The campaign can be understood as a form of communication that involves verbal and non-verbal elements. It allows a message to be effectively conveyed through images and videos to an audience. Generally, the term ‘campaign’ is often associated with political communication, especially during elections. Charles U. Larson classifies campaigns into several types based on their objectives: (1) candidate-oriented, which focuses on the candidate; (2) product-oriented, which emphasizes the product or service; and (3) ideologically-oriented, which promotes a particular ideology or cause. The ideologically-oriented campaigns, such as campaigns to moderate religion, are considered to address social problems such as changes in people's behavior, attitudes, and views [54].

1. Muhammadiyah's Campaigns

Muhammadiyah actively promotes religious moderation through its official Instagram account, sharing posts that emphasize tolerance, community service, and interfaith dialogue. Their campaigns often highlight educational programs aimed at youth to foster understanding and respect among different religious groups.

2. Ministry of Religion Initiatives

The Indonesian Ministry of Religion utilizes its Instagram platform to disseminate messages of harmony and moderation in Islam. They conduct campaigns that focus on promoting national unity and the importance of respecting diverse beliefs, often featuring testimonials from various community leaders.

3. Moderate Islamic Influencers

Various influencers on Instagram advocate for religious moderation by sharing personal stories and experiences that promote peaceful coexistence and understanding among different faiths. They often engage their followers through interactive content, such as Q&A sessions and live discussions about tolerance and acceptance.

These examples will strengthen the literature review by demonstrating the broader efforts in promoting religious moderation on social media platforms beyond NU Online.

Ideological campaigns should be strengthened by including symbolic engagement in their activities. Mead introduced the concept of symbolic interaction, which refers to three basic concepts: (1) the mind, which allows individuals to organize symbols with the same social meaning, so that each person develops his or her own perspective when interacting with others; (2) the personal self, which includes the individual's ability to understand himself or herself from the perspective of others; and (3) the community, which describes the interactions organized by individuals [55]. As a result, Instagram users discovered a variety of religious moderation campaigns' content using the hashtag #moderasiberagama. It is evidenced by the diversity of Instagram users who propagate the idea of religious moderation through the different contents they publish, such as the following:

The Instagram account @nuonline_id promotes religious moderation through posters and short videos that incorporate religious moderation concepts. The campaign message is critical to its success. The Instagram account @nuonline_id belongs to Nadhlatul Ulama (NU), Indonesia's largest Islamic organization, with ulama and santri members spread across the country. In 2023, Nadhlatul Ulama will enter the second century of its quest for national unity, integrity, and safety. Nadhlatul Ulama follows Ahlussunnah wal Jama'ah, which uses the madhhab method to comprehend, live, and apply Islamic principles. Nadhlatul Ulama believes that adhering to a defined mazhab helps members of the organization stay on the right track and attain the genuine teachings of Islam.

The Instagram account @nuonline_id explains that Indonesia is a country rich in ethnic, national, and religious diversity. The Instagram account actively campaigns for religious moderation and emphasizes the important role of scholars and academics in this campaign. Religious moderation messages include all forms of communication, both verbal and nonverbal. Verbal communication means communication through speech, while nonverbal communication involves gestures, touch, smell, feelings, and symbols. There are three main factors in conveying messages, namely: (1) message material, (2) message form, and (3) message code.

The @nuonline_id account has promoted religious moderation through posters and short videos with messages and symbols related to religious moderation. The symbols include various places of worship and religious dress, which represent Indonesia's religious variety. The message to be conveyed is explained in the post's caption. The images in the account post depict tolerance and mutual respect among religious communities. Furthermore, hadith citations in conformity with Islamic teachings demonstrate that Islam opposes bigotry, prejudice, and violence. It demonstrates how Islam promotes peace and harmony among religious communities.

The campaign's message is a persuasive appeal intended to increase human understanding and awareness of the message's content, which has the ability to influence someone's mind. The message has been elegantly produced in the form of posters and short movies to pique the curiosity of other users. As a result, a vast number of people are exposed to the messaging, which promotes comprehension and awareness and is expected to improve people's attitudes about religious moderation.

Nadhlatul Ulama (NU) is entering its second century in 2023, marking a significant milestone for Indonesia's largest Islamic organization. As NU continues its commitment to national unity, integrity, and safety, it emphasizes the importance of religious moderation through various campaigns. The organization actively promotes these values on social media platforms, particularly Instagram, where its account @nuonline_id shares content that highlights the significance of tolerance and respect among Indonesia's diverse religious communities.

In addition to the efforts of NU Online, there are other examples of religious moderation campaigns that utilize social media effectively. Various organizations and community groups have launched initiatives promoting understanding and coexistence among different faiths. These campaigns often employ engaging visuals and relatable messaging to reach a wider audience, encouraging dialogue and fostering a culture of moderation. By showcasing diverse perspectives and emphasizing common values, these campaigns contribute to a broader movement advocating for peace and harmony in society.

## 6 Conclusion

Promoting religious moderation in Indonesia is essential for fostering unity among diverse faiths and maintaining social stability. This study explores how the Quran's teachings, particularly the principle of *wasathiyyah*—a balanced and moderate approach to religious practice—can inspire campaigns on social media platforms like Instagram. Religious moderation is both a theoretical framework and a practical imperative, emphasizing mutual respect, acceptance, and coexistence among different faith communities. By adopting a balanced attitude toward diverse perspectives, religious moderation not only reduces conflicts but also strengthens the foundation for a harmonious and pluralistic society.

Effective implementation of religious moderation campaigns on Instagram requires strategic approaches that resonate with a digitally engaged audience. Engaging content, such as persuasive visuals, informative videos, and interactive discussions, plays a pivotal role in promoting the values of moderation. Hashtags like #moderasiberagama enhance visibility and foster a sense of community, while Qur'anic principles such as mutual respect (*tasamuh*), balance (*tawazun*), and justice (*i'tidal*) are translated into impactful content. Instagram's dynamic features enable open dialogue and active participation, making the message of moderation both relatable and impactful for broader audiences.

Focusing on socialization, community education, and fostering dialogue through social media aligns with the core values of the Qur'an and addresses the pressing need for religious moderation in Indonesia. This study highlights the potential of Instagram-based campaigns in countering radical ideologies and promoting inclusivity. However, the exclusive focus on Instagram and the lack of quantitative analysis of user interactions, such as comment responses, present limitations. Future research should expand to multiple social media platforms and incorporate quantitative methodologies to provide a more comprehensive understanding of digital media's role in fostering religious harmony and addressing Indonesia's complex religious landscape.

## CRediT authorship contribution statement

**Andy Hadiyanto:** Writing – original draft, Resources, Methodology, Data curation, Conceptualization. **Kinkin Yuliaty Subarsa Putri:** Writing – original draft, Resources, Methodology, Data curation, Conceptualization. **Luthfi Fazli:** Writing – original draft, Resources, Methodology, Data curation, Conceptualization.

## Ethical approval

This study has received formal approval from the relevant institutional authority, confirming compliance with established ethical standards. The research protocol was reviewed and approved prior to data collection, ensuring adherence to institutional and international ethical guidelines. No human participants were involved in this study, and all data sources were obtained through ethical and legitimate means.

## Data availability statement

Data included in the article/supplementary material is referenced in the article.

## Declaration of competing interest

The authors declare that they have no known competing financial interests or personal relationships that could have appeared to influence the work reported in this paper.
